# Schüler*innenprotest – subversive Praxis oder Einlösung schulischer Partizipationsversprechen?

**DOI:** 10.1007/s42278-021-00110-1

**Published:** 2021-07-09

**Authors:** Torsten Eckermann

**Affiliations:** Institut für Erziehungswissenschaft, Europa-Universität Flensburg, Flensburg, Deutschland

**Keywords:** Schüler*innenprotest, Partizipation, Grundschule, Rekonstruktive Analyse, Student protest, Participation, Elementary school, Reconstructive analysis

## Abstract

Im Rahmen des Beitrags werden Ergebnisse aus einem laufenden ethnographischen Forschungsprojekt zur Schüler*innenprotestbewegung „Fridays-for-Future“ präsentiert. Erste rekonstruktive Analysen deuten dabei darauf hin, dass aus Sicht der protestierenden Schüler*innen der Grundschule als Bildungsinstitution eine besondere Relevanz für die Etablierung klimaprotektiver Praktiken zukommt, wobei vor allem Grundschulen mit einem spezifischen Profil („Draußenschule“, „Klimaschule“) adressiert werden. Gleichwohl ein dezidiert schuloppositionelles Verhalten nicht zu identifizieren ist, sondern die Schüler*innenprotestbewegung die Grundschule eher als ‚verlängerten Arm‘ der politischen Agenda entwirft, bedienen sie sich subversiver Praktiken, indem sie punktuell die gesetzliche Schulpflicht außer Kraft setzen. Der Beitrag lenkt den Fokus auf die bislang vernachlässigte Partizipation von Grundschüler*innen an den Protestpraktiken aus der Sicht jugendlicher Aktivistinnen und nimmt dabei in den Blick, wie sich der Schüler*innenprotest zur Schule ins Verhältnis setzt.

## Abstract (en)

This paper presents results from an ongoing ethnographic research project on the student protest movement “Fridays for Future”. The reconstructive analysis of data shows that, from the perspective of the protesting students, the elementary school (especially with a specific profile “outdoor school”, “climate school”) is of particular relevance for the establishment of climate-protective practices. Although a school-oppositional behavior cannot be identified, but the student protest movement rather considers the elementary school as an ’extended arm’ of the political agenda, they make use of subversive practices by selectively suspending the legal compulsory education. This article focuses on the neglected participation of elementary school students in protest practices from the perspective of young activists and examines how student protest relates to school.

## Einleitung

Auf der Grundlage der UN-Kinderrechtskonvention hat Krappmann (2014, S. 19) in pointierter Weise herausgestellt: „Für Schule gibt es keinen demokratiepädagogischen Ersatz“. In dem von ihm formulierten Kinderrechte-Manifest betont er, dass Schule ein Ort sei, „der Kindern und Jugendlichen am menschlichen Wohl orientiertes, demokratisches Zusammenleben erlebbar und einsichtig machen“ (Krappmann 2016, S. 26) könne. Bildungsinstitutionen wie der Grundschule käme demnach die besondere Relevanz zu, Kinder mit demokratiebildenden Verfahren (z. B. konsensuelle Mehrheitsentscheidungsfindung) und demokratischen Praktiken (z. B. Urteilsbildung, freie Meinungsäußerung) vertraut zu machen. Voraussetzung dafür sei, dass die Grundschule den Kindern eine „demokratische Beteiligungskultur“ (Krappmann 2016, S. 41) biete, etwa in Gestalt des Klassenrats oder anderer institutionalisierter Mitbestimmungsgremien wie dem Schüler*innenparlament oder der Schüler*innenvertretung. Die Grundschule sei dabei – wie auch Dewey (1964) betont – als demokratische Lebensform zu entwerfen, in der Demokratie nicht nur angekündigt, sondern auch praktiziert werde (ebd., S. 47).

Den Entwurf zu einer solchen „demokratischen Grundschule“ diskutieren auch Backhaus et al. (2008). Die Autor*innen benennen dabei nicht nur Potenziale, sondern markieren zudem wesentliche Herausforderungen, die eine partizipative Ausgestaltung des Unterrichtsalltags und eine demokratische Schulkultur implizieren. Kritisch hinterfragt wird dabei, inwieweit sich Grundschullehrkräfte und Schüler*innen tatsächlich als demokratisch Handelnde und Mitbestimmende und nicht nur „als von Weisungen Abhängige“ (Backhaus et al. 2008, S. 32) erlebten; etwa deshalb, weil eine demokratische Abstimmung darüber, was im Unterricht gelehrt und gelernt werde, in der Regel ausbleibe, da es durch staatliche Vorgaben reglementiert sei. Neben den limitierten Partizipationsmöglichkeiten bei den Unterrichtsinhalten problematisieren die Autor*innen, dass in der pädagogischen Praxis der Grundschule mitunter ein spezifisches Verständnis von Partizipation kultiviert werde, wobei die Lehrkräfte definierten, „was Partizipation ist, wie diese auszusehen hat und wann diese gefragt ist“ (ebd.). Damit fehle es an der notwendigen Sensibilität für die „Formen von Mitbestimmung, die die Kinder selbst wählen“ (ebd.).

Aus den skizzierten Einwänden lässt sich entnehmen, dass die umfassenden Demokratisierungsbestrebungen der Grundschule – trotz des Ausbaus an partizipatorischen Mitbestimmungsoptionen im schulischen Kontext – an Grenzen stoßen, was einerseits mit der terminologischen Unschärfe des Partizipationsbegriffs und den überhöhten Erwartungen an das, was mit Partizipation bewirkt werden soll, korrespondiert (2.1), andererseits auf schulstrukturelle Bedingungen zurückzuführen ist (2.2). Beiden Aspekten soll sich nachfolgend gewidmet werden. Jenseits des institutionellen Kontexts der Schule wird sich zudem der Partizipation der Grundschüler*innen an den Protestpraktiken der Schüler*innenprotestbewegung zugewandt. Dabei soll das Spannungsverhältnis zwischen den pädagogisch motivierten Ansprüchen innerschulischer Partizipationsformate und der außerschulischen Partizipation an den Protestpraktiken ausgeleuchtet werden (3.). Zurückgegriffen wird dazu auf empirisches Material aus einem laufenden ethnographischen Forschungsprojekt. In einem resümierenden Ausblick diskutiert der Beitrag das Verhältnis der Politisierung (grundschul-)pädagogischer Praxis und die schulische Externalisierung des Schüler*innenprotests (4.).

## Partizipation von Schüler*innen und die „Grammatik der Schule“

### Zum Ruf nach (mehr) Partizipation im Lichte empirischer Befunde

Ein Blick in die Fachliteratur verdeutlicht, dass der Begriff „Partizipation“, der in der (reform-)pädagogischen Tradition mit Korczak, Dewey oder auch Freinet eine lange Historie aufweist, wieder Konjunktur hat (vgl. Billis 2020; Gerhartz-Reiter und Reisenauer 2020; Rieker et al. 2015). So diagnostizieren Rieker et al. (2015, S. 7) einen regelrechten „Partizipationsboom“ und Reichenbach (2006, S. 39) spricht von einer „Partizipationseuphorie“. Den Partizipationsbegriff zeichnet dabei aus, dass er positiv konnotiert ist und ihn ein „mythischer Mantel allheilbringender Wirksamkeitskraft“ (Biedermann und Oser 2010, S. 28) umgibt. Im schulpädagogischen Diskurs wird Partizipation bisweilen als „Allheilmittel“ (Reichenbach 2006, S. 58) angepriesen und der Ruf nach mehr Partizipation – hergeleitet aus demokratietheoretischen, bildungstheoretischen oder menschenrechtlichen Erwägungen – erzeugt nur selten Widerspruch (vgl. Olk und Roth 2007). Vereinzelte Studien untermauern die positiven Wirkungen: So würden sich Schüler*innen im Unterricht eher beteiligen, wenn sie in die Unterrichtsplanung einbezogen werden (Howley und Tannehill 2014); zudem ließen sich durch ein reichhaltiges Angebot an Beteiligungsmöglichkeiten ihre sozialen sowie kommunikativen Fähigkeiten verbessern (vgl. Mayes et al. 2019). Aus den Ergebnissen dieser Studien lässt sich der Eindruck gewinnen, dass das Partizipationsversprechen in der Grundschule uneingeschränkt eingelöst wird. Allerdings geht aus anderen Studien wie dem DJI-Kinderpanel – hervor, dass Grundschulkindern zwar Mitbestimmungsmöglichkeiten im Hinblick auf Pausenregeln, Sitzordnung oder Klassenraumgestaltung eingeräumt bekommen, allerdings weit weniger beteiligt werden, wenn es um Entscheidungen über Unterrichtsinhalte oder die Leistungsbewertung geht (Weber et al. 2008). Dies deckt sich auch mit anderen empirischen Befunden, die aus der Ganztagschulforschung stammen, wonach Kinder und Jugendliche ihre Partizipationsmöglichkeiten umso größer einschätzen, je weiter die Bereiche sich inhaltlich vom Unterricht entfernen (vgl. Billis 2020, S. 373). Auch die Studie von Oser und Biedermann (2007, S. 25) verweist darauf, dass die Schüler*innen außerhalb der Schule, d. h. in der Familie, signifikant mehr mitbestimmen können als in der Schule, und dies „selbst bei Dingen, die prima facie ihr Leben betreffen“. Es drängt sich somit der Verdacht auf, dass die Grundschule, Partizipationsräume eröffnet, die auf spezifische *innerschulische* Bereiche limitiert sind.

Bislang erweist sich die Forschungslage zur Partizipation von Kindern im Grundschulalter als eher dünn (vgl. Coelen und Wagener 2011). Hinzu kommt, dass aufgrund der Offenheit des Partizipationsbegriffs und seiner heterogenen Verwendung häufig nebulös bleibt, „unter welchen Bedingungen was für Formen von Partizipation realisiert werden“ (Biedermann und Oser 2010, S. 28). Dies ist insbesondere vor dem Hintergrund relevant, dass in der Grundschule ein breites Spektrum an Beteiligungsformaten besteht, die sich auf folgende Bereiche beziehen:

- *selbstverantwortliche Organisation des Lernens* (z. B. mit Hilfe des Wochenplans)
- *klassenöffentliche Mitbestimmungsgremien* (z. B. den Klassenrat)
- *die Übernahme von Ämtern* (z. B. Klassensprecher*in, Schüler*innenvertretung)
- *auf projektförmige Beteiligungsformen* (z. B. zur Schulhofgestaltung)

Innerhalb dieser Beteiligungsformate können zudem die Partizipationsintensitäten variieren. Es ist demnach stets eine empirische Frage, „wie selektiv oder inklusiv, unverbindlich oder nachhaltig, dauerhaft oder kurzfristig Beteiligung angelegt ist“ (Olk und Roth 2007, o. S.).

Bilanzierend lässt sich feststellen, dass es sich beim Partizipationsbegriff um einen Begriff mit „verschwommenen Rändern“ (Wittgenstein 1967, S. 50) handelt, der synonym zu Begriffen wie Teilhabe oder Mitbestimmung Verwendung findet, obgleich zweitere eine andere Qualität (i. S. stärkere *aktive* Beteiligung) – impliziert als erstere, die auf ein Recht verweist, das eingelöst werden kann oder nicht (Rieker 2017, S. 316). Der Begriff wird bisweilen normativ überformt, sodass „Partizipation an sich etwas Gutes sein müsse“ (Oser und Biedermann 2007, S. 25), womit jedoch die Unterrichtswirklichkeit oftmals dem Ideal der gelungenen Partizipation hinterherhinkt. Für Oser und Biedermann (2007, S. 19) stellt die begriffliche Konfusion mit anderen Begriffen und die Verwässerung des Begriffs ein zentrales Problem dar, da der Begriff nur dann als pädagogischer Begriff taugt, wenn nicht einfach vorausgesetzt wird, dass Partizipation etwas ist, was man immer schon *kann* und alle Schüler*innen gleichermaßen uneingeschränkt *wollen*. Eine für alle erzwungene pädagogisch motivierte Partizipation führt nicht zu vermehrter Egalität, sondern „vielmehr häufig zu vermehrter Subtilität der alten und neuen Machtverhältnisse“ (Reichenbach 2006, S. 58).

### Pädagogisierung der Partizipation – Zwischen Autonomie und Heteronomie

Gleichwohl sich demokratiepädagogisch und bildungstheoretisch argumentierend gute Gründe anführen lassen, warum Partizipation als zentraler Bestandteil demokratischen Handelns bereits früh in der Kindertagesstätte (vgl. „Kinderstube der Demokratie“: Hansen et al. 2009; Prengel 2016) und in der Grundschule (vgl. Burk et al. 2003; Brügelmann 2014) ein Bildungsziel darstellt, so offenbart sich in der Schul- und Unterrichtspraxis, dass sich die Realisierung häufig nicht wie erhofft einstellt (vgl. Leser 2011). In seiner Expertise zum 16. Kinder- und Jugendbericht führt Brügelmann (2020) als mögliche Gründe dafür an, dass im Sachunterricht, dem als Unterrichtsfach eine wesentliche Verantwortung für die Demokratiebildung in der Grundschule zukäme, die Anbahnung naturwissenschaftlicher, historischer und geographischer Bildung im Vordergrund stehe. Zudem friste die Didaktik politischer Bildung „in der Grundschule ein Nischendasein“ (ebd., S. 2) und institutionalisierte Mitbestimmungsgremien seien weiterhin unterentwickelt (S. 26 ff.). Letzteres lässt sich dabei jedoch weniger auf eine Partizipationsunwilligkeit der Schüler*innen oder einer Partizipationsblockadehaltung seitens der Lehrer*innen zurückführen, vielmehr ist die Schüler*innenpartizipation verstrickt in die pädagogische Handlungsantinomie von Autonomie und Heteronomie, woraus paradoxe Anforderungen an das Lehrer*innen- und Schüler*innenhandeln resultieren (vgl. Helsper und Lingkost 2004). So soll mit der Aufforderung zur Partizipation den Schüler*innen zu größerer Autonomie verholfen werden, ohne dass dabei allerdings die „Grammatik der Schule“ (Tyack und Tobin 1994) – wie beispielsweise die gesetzliche Schulpflicht, die limitierten Aushandlungsspielräume der Schüler*innen bei der Leistungsbewertung, der Gestaltung des Stundenplans oder der fehlenden Möglichkeit, über Lehrkräfte mitentscheiden zu dürfen – außer Kraft gesetzt wird. Die Schüler*innenpartizipation kann damit zu einer inszenierten, simulierten und verordneten Autonomie werden (vgl. Helsper und Lingkost 2004; Budde 2010). Das Partizipationsideal, die Grundschule im Sinne der antiken „*polis*“ zu entwerfen, wird vor diesem Hintergrund konterkariert. Wie bereits Reichenbach (2006) diagnostiziert, können „Schulen *nie nur* demokratisch sein … [womit sich] die Begrenztheit aller politischen Schulbildung andeutet“. Das schulische Partizipationsversprechen konfligiert mit den institutionellen Logiken der Schule – und dies nicht zuletzt auch deshalb, da der Schule die Funktion zukommt, bestehende „gesellschaftliche Herrschaftsverhältnisse und Verteilungsprozesse zu legitimieren“ (Leser 2011, S. 1). Damit besteht allerdings der Verdacht, dass die Partizipation in der Schule dort endet, wo der Protest gegen die schulischen Logiken und Ordnung beginnt. Die Limitiertheit der Partizipation im Kontext der (Grund‑)Schule zeigt sich auch mit Blick auf die Möglichkeit zur Artikulation von Protest, der als spezifische, radikale Partizipationsform weitgehend exkludiert wird. Denn obwohl Grundschüler*innen mit Hilfe von institutionalisierten Partizipationsformaten einen demokratischen Habitus erwerben sollen, der auch über die Schule hinaus Bestand hat (Edelstein 2007), werden die partizipativen Praktiken nicht mit dem Ziel eingesetzt, die schulische Ordnung selbst in Frage zu stellen und Grundkonflikte des Bildungssystems sichtbar zu machen. Es lässt sich somit der Eindruck gewinnen, dass Partizipation in der Schule in diesem Sinne zuerst vor allem der Einübung in und dem Erlernen von Partizipation *in* der Schule dient (vgl. Wigger et al. 2019).

Genau hier knüpft das nachfolgend vorzustellende ethnographische Forschungsprojekt an und fragt jenseits des institutionellen Kontexts der Schule: Wie partizipieren Grundschüler*innen an Protestpraktiken jenseits des institutionellen Kontexts der Schule? Und wie wird sich dabei auf die (Grund‑)Schule als Bildungsinstitution bezogen?

## Partizipation durch Protest? Ethnographische Einblicke in die Schüler*innenprotestbewegung „Fridays-for-Future“

Nachfolgend sollen Ergebnisse aus einem laufenden ethnographischen Forschungsprojekt „Schule im (Klima‑)Wandel? – Eine ethnographische Studie zur Schüler*innenprotestbewegung Fridays-for-Future“ präsentiert werden^1^. Im Rahmen des Projekts, welches im Januar 2020 begonnen hat, also ein Jahr nachdem die global organisierte Fridays-for-Future-Bewegung in Deutschland bereits an 50 Orten demonstrierte, werden einerseits teilnehmende Beobachtungen entlang der Demonstrationsrouten durchgeführt und audiogestützte Feldinterviews mit den Teilnehmer*innen vor Ort geführt (vgl. Breidenstein et al. 2013). Nachdem sich aufgrund der COVID-19-Pandemie der Protest der Bewegung vermehrt in digitale Räume verschoben hat, wurden leitfadengestützte Einzelinterviews mit 15 Schüler*innen (Telefoninterviews oder per Videokonferenztools) durchgeführt. Die Interviews dienen einerseits dazu, mehr über die Motivation der Schüler*innen sich an den On- und Offline-Protesten zu beteiligen zu erfahren, andererseits ist von Forschungsinteresse, inwieweit die Proteste Gegenstand der Gespräche in der Schule mit Lehrkräften und zu Hause mit den Eltern sind. Während mit Hilfe der teilnehmenden Beobachtung vor allem die tatsächlichen Protestpraktiken (bzw. das „Wie“ des Protestierens) im Vordergrund stehen, werden mit den Interviews Narrationen über die Zugehörigkeit zu der Protestbewegung generiert. Um das empirische Material in einem ersten Schritt ‚aufzubrechen‘, wurde mit Hilfe der Grounded-Theory-Methodology (GTM, Strübing 2014) zunächst offen kodiert und im Anschluss daran erfolgte eine extensive sequenzanalytische Auswertung (vgl. Meseth 2013). Für die Auswahl des hier präsentierten empirischen Materials wurde als zentrales Kriterium herangezogen, dass sich die Aussagen auf die Grundschule beziehen, um das Verhältnis zwischen Protestpraktiken und (Grund‑)Schule zueinander relationieren zu können^2^. Das ausgewählte Material zielt dabei auf die „individuelle Allgemeinheit des Falles“ (Bude 1985) ab, d. h. an ihm werden zentrale, fallübergreifende Momente des Schüler*innenprotests sichtbar.

### Projektionen von Grundschule: Die Zukunft beginnt in der Grundschule

Die vorliegenden Analysen verweisen darauf, dass die befragten Schüler*innen im Alter zwischen 12 und 16 Jahren auf die Frage nach ihrer Motivation, sich bei Fridays-for-Future und für den Klimaschutz zu engagieren, auf die Grundschulzeit als bedeutsamen zeitlichen Marker rekurrieren. So teilt uns eine der Befragten im Interview mit:

Helena: „… ja, also man merkt das schon in der Grundschule. meine Mutter unterrichtet ja an der Stadtschule und ehm die sind ja auch Klimaschule und haben diese Draußenschule als Aktion ehm … und dann merkt man halt schon, dass die Kinder da halt mehr drüber nachdenken und dann halt auch die Sachen, die ihre Eltern machen halt hinterfragen … also warum sie zum Beispiel jeden Tag zur Schule gefahren werden obwohl es nur fünf Minuten sind und sowas alles. und man merkt halt schon, wenn wir das Thema im Unterricht behandeln, dass mehr Leute dann auch drüber nachdenken und zum Beispiel jetzt Mitschüler von mir haben jetzt statt immer ’ne Plastikflasche dabei zu haben jetzt auch mal ’ne Metallflasche dabei oder kaufen nicht mehr obst, das immer das in Plastik soviel eingeschweißt ist und so also sowas nennt man halt schon so kleine Veränderungen.“ #00:06:28#

In diesem Interviewausschnitt dokumentiert sich exemplarisch, dass aus Sicht von *Helena* die Grundschule bei Kindern wesentlich Einfluss nehmen könne auf die Fähigkeit, das eigene klimaprotektive bzw. -bedrohende Verhalten sowie das der Eltern zu reflektieren. Als Beispiel für die von der Grundschule ausgehende Sensibilisierung benennt sie einen spezifischen Aspekt von Mobilität (Autofahren zur Schule), der auch in Zusammenhang mit den Forderungen zur Reduktion der CO2-Emissionen der Schüler*innenprotestbewegung steht (vgl. Sommer et al. 2019). Auffällig ist, dass Helena mit dem Autofahren zur Schule auf eine für Grundschüler*innen etablierte Alltagsroutine referiert, die es – sofern es sich um kürzere Strecken handelt – im Sinne der Schüler*innenprotestbewegung zu durchbrechen gelte. Damit positioniert sich Helena hier als Fürsprecherin der Protestbewegung, zeigt ihre Zugehörigkeit an und legitimiert den Schüler*innenprotest insofern, als dieser auf eine stärkere Fortschrittlichkeit und Zukunftsgewandtheit („For Future“) sowie eine Transformation etablierter Alltagspraktiken abzielt (vgl. Sommer et al. 2019). Weiterhin fällt an Helenas Ausführungen auf, dass Kindern im Grundschulalter eine Handlungsmächtigkeit (Agency) attestiert wird, die sich darin ausdrückt, klimagefährdende elterliche Praktiken zu hinterfragen. Darin kommt einerseits zum Ausdruck, dass die Kinder explizit als Partizipanden der Schüler*innenprotestbewegung adressiert werden; zum anderen zeigt sich eine spezifische Form des *generationalen Umordnens*, d. h. sie werden mit Blick auf den ‚ökologischen Fußabdruck‘ der Eltern als Kritik‑, Kontroll- und Korrekturinstanzen entworfen^3^. Die Elterngeneration dient in diesem Sinne als Abgrenzungsfolie, daraus folgt jedoch keine fundamentale Oppositionshaltung gegenüber ‚Autoritäten‘ wie den Bildungsinstitutionen. Im Gegenteil: Grundschulen mit einem spezifischen pädagogischen Profil („*Draußenschule*“^4^, „*Klimaschule*“), werden als ‚Komplizen‘ zur Umsetzung der politischen Ambitionen der Schüler*innenprotestbewegung betrachtet.

Analog zu Helena sieht auch die Schülerin *Lilly* das besondere Potenzial in der Grundschule darin, Themen, die für die Schüler*innenprotestbewegung relevant erscheinen, zu institutionalisieren:

Lilly: „Ja man sollte da früh anfangen. Ich hab da auch nie was zu gemacht. Auch nicht in der Grundschule. Was hat das zum Beispiel für Auswirkungen auf die Welt, wenn wir nicht Mülltrennen. Sowas kann man auch in der Grundschule machen finde ich.“

Auch aus den Schilderungen Lillys geht hervor, dass die Grundschule als Institution ein zentraler Referenzpunkt ist: So sei es sinnvoll, das Thema Klimawandel im Besonderen und den Umweltschutz im Allgemeinen möglichst frühzeitig in der Grundschule zu verankern, was allerdings in ihrer eigenen Schulbiographie ausgeblieben sei (*„Ich hab da auch nie was zu gemacht“*). Mit ihrer Aussage verweist sie auf die Notwendigkeit, dass die Grundschule sich dieser Aufgabe annehme, ohne dass sich dies durch ihre eigenen schulbiographischen Erfahrungen aus der Grundschulzeit deckt. Sie exemplifiziert die Möglichkeit der Vermittlung im Unterricht an einem Thema des Alltags, welches zugleich fester Bestandteil des Sachunterrichts ist – der „Mülltrennung“. Lillys Äußerung „Sowas kann man auch in der Grundschule machen finde ich“ lässt sich als Indiz dafür lesen, dass die Mülltrennung neben anderen Themen auch seinen Platz im Grundschulunterricht finden sollte. Deutlich wird in der Aussage, dass das Thema Klimawandel für die protestierenden Schüler*innen nicht nur auf die politische Agenda gesetzt werden, sondern seinen curricularen Niederschlag auch in den Bildungsinstitutionen wie der Grundschule finden müsse.

Die Bemerkungen von Helena und Lilly deuten darauf hin, dass der Grundschule eine Relevanz bei der Reflexion und Einübung klimaprotektiver Praktiken attestiert wird, obgleich beide Schülerinnen nicht explizit aus eigenen schulbiographischen Erfahrungen ihrer Grundschulzeit berichten. Sie projizieren stattdessen Erwartungen an die Institution Grundschule und an die Grundschüler*innen als Akteure, die aus ihrer Sicht einen Beitrag zur Transformation etablierter Alltagspraktiken hin zu einer nachhaltigen Gesellschaft leisten können. Veranschaulicht wird dies an Alltagspraktiken (z. B. mit Auto zur Schule fahren), die zugleich spezifisch ausgewählte Unterrichtsthemen (Mülltrennung) sein können; gleichzeitig deutet sich an, dass Kinder im Grundschulalter als Partizipanden der Schüler*innenprotestbewegung adressiert werden, worauf nachfolgend näher eingegangen werden soll.

### Partizipation der Grundschüler*innen am Protest

Bei unseren teilnehmenden Beobachtungen der Demonstrationen im Raum Schleswig-Holsteins konnten wir immer wieder beobachten, dass Grundschüler*innen direkt am Kopf des Demonstrationszuges gehen. Ausgestattet sind sie dabei mit ihren zu Hause angefertigten oder auch von dem jeweiligen Organisationsteam der Fridays-for-Future-Schüler*innenprotestbewegung überreichten Frontbanner. Auf die freitäglichen Demonstrationen kommen sie mit Klassenkameraden, mitunter auch mit ihren Eltern. Zur Partizipation der Grundschüler*innen am Protest berichtet uns *Julia* etwa folgendes:

Julia: „Ehm also wir haben auf unserer Demo haben wir immer … ehm ungefähr so ne ganze Klassen von Grundschülern die dann auch immer vorne unsren Frontbanner halten und super mitjubeln und ehm … das ist halt immer ziemlich süß das so zu sehen wie die sich auch engagieren das freut einen natürlich auch … und dann gehen wir halt manchmal mit dem Megafon dahin und fragen sie ob sie nen Spruch rufen möchten und dann hören wir immer sehr oft ‚Bäume sind Freunde‘ und unterhalten uns auch immer im Orgateam darüber wie wir diesen Spruch finden und kommen eigentlich immer zu dem Fazit, dass wir das super toll finden wenn die das rufen.“

In diesem Interviewausschnitt verweist Julia darauf, dass neben den älteren Schüler*innen auch Grundschüler*innen an den Freitagsdemonstrationen teilnehmen. Diese würden an den Protestpraktiken partizipieren, indem sie „unsren Frontbanner tragen“, „super mitjubeln“ und gelegentlich auch durch das Megaphon „nen Spruch“ wie „Bäume sind Freunde“ skandieren. Anhand dieser Passage deutet sich an, dass politisches Engagement und der Eventcharakter beim Protest auf der Straße verschwistert sind. Dieser Eventcharakter hat der Schüler*innenprotestbewegung in der medialen Öffentlichkeit den Vorwurf eingebracht, dass es ihnen nicht primär um die Artikulation politischer Interessen, sondern um das Schule schwänzen gehe – worauf später zurückzukommen sein wird. Die Anzahl der Grundschüler*innen, die sich an den Demonstrationen beteiligen, wird von Julia grob quantifiziert („so ne ganze Klassen“), was vermutlich darauf hindeuten soll, dass es sich nicht nur um einzelne, sondern eine größere Anzahl an Grundschüler*innen handelt. Entscheidend ist dabei jedoch nicht die reine Quantität, sondern die *Platzierung* der Grundschüler*innen im Kontext des Demonstrationszugs („..*immer vorne*“). Da soziale Bewegungen mit ihren politischen Aktionen (z. B. Sit-Ins) oftmals auch das Anliegen verfolgen, auf die Dynamisierung und Neuinterpretation des gesellschaftlichen Raums zu verweisen und dazu die Straße als öffentlichen Raum nutzen (Maurer 2018), sind auch die Gruppenformationen innerhalb des Demonstrationszugs relevant. Die Platzierung der Grundschüler*innen mag unter pragmatischen Gesichtspunkten funktional sein, da sie die Aufgabe zugewiesen bekommen, das Frontbanner zu tragen; andererseits ist diese Platzierung auch prestige- und symbolträchtig, da sie den Demonstrationszug anführen. Am Kopf des Demonstrationszugs zu gehen, hat eine hohe Symbolkraft, da die jüngeren Grundschüler*innen – ganz im Sinne des Namens der Schüler*innenprotestbewegung – besonders prädestiniert erscheinen, die Zukunft zu repräsentieren. Zugleich deuten die Aussagen der Schülerin auf einen *ambivalenten Partizipationsstatus* der Grundschüler*innen im Rahmen der Protestbewegung hin: So werden die Protestpraktiken der Grundschüler*innen als „ziemlich süß“ klassifiziert. Die Attribuierung „süß“ steht dabei nicht nur im Widerspruch zu dem, was man für gewöhnlich mit Protest als oppositionelles Verhalten assoziiert, sie lässt auch die Protestpraktiken der Kinder als (politisch) nicht ernstzunehmend erscheinen. Diese Differenzierung entlang des Alters und der Jahrgangsstufen der Kinder expliziert Julia in einer weiteren Interviewpassage:

Julia: „Also ich würd sagen dass man sie so ab dritte Klasse auch mal alleine sieht … aber so die kleineren kommen meistens mit ihren Lehrern und das ist halt hier bei uns auch dass die Lehrer dann wirklich mitkommen … also eigentlich ist immer nur eine Klasse da aber das freut einen natürlich schon immer dass man dann auch sieht die Schulen unterstützen das dann auch.“

Die Bemerkungen Julias verdeutlichen in diesem Interviewausschnitt erneut die ‚Komplizenschaft‘ zwischen den Repräsentant*innen der Schule („mit ihren Lehrern“) und der Schüler*innenprotestbewegung. Im Kontrast dazu steht jedoch, was uns Hannah während einer Demonstration erzählt:

Hannah: „eh in der Schule spreche ich auch mit meinen Lehrern darüber die meisten von meinen Lehrern sind halt dagegen und sagen so es gibt den Klimawandel nicht ähm

I: ok

Hannah: das ist bescheuert dass du da hingehst und vor allem Greta Thunberg sie hatte keine schwere Kindheit die Kinder im Krieg haben ne schwere Kindheit sagen also echt komische Sachen eh aber das macht mir halt nichts also einige sind dann echt unfair zu mir

I: ok

Hannah: deswegen würde ich auch gern die Schule wechseln … und die Klassenkameraden von mir die sagen halt es ist unnatürlich Vegetarier zu sein und … es ergibt keinen Sinn dass du dahin gehst weil es gibt keinen Grund dazu also.“

Die Aussagen Hannahs verweisen zunächst darauf, dass der Schüler*innenprotest auch bei ihr in der Schule aufgegriffen wird und Bestandteil der Gespräche mit Lehrkräften ist. Wie sie ausführt, seien jedoch „die meisten von meinen Lehrern“ gegen den Schüler*innenprotest und würde den anthropogenen Klimawandel leugnen („es gibt den Klimawandel nicht“). Im Kontrast zu den vorausgegangenen Aussagen der Schülerinnen, nach denen Grundschullehrkräfte an den Freitagsdemonstrationen teilnehmen und die Schüler*innen dorthin begleiten, dokumentiert sich hier eine explizit ablehnende Haltung der Lehrkräfte. Die Kritik an der Schüler*innenprotestbewegung entzündet sich offensichtlich auch an der schwedischen Klimaaktivistin Greta Thunberg, die als Initiatorin der Schüler*innenprotestbewegung gilt. An den Schilderungen Hannahs wird jedoch deutlich, dass es nicht nur die Lehrkräfte sind, die Vorbehalte gegenüber der Schüler*innenprotestbewegung formulieren, sondern auch die Mitschüler*innen. Von diesen würden sie etwa für ihr Ernährungsverhalten kritisiert werden. Auch wird ihr in ganz grundsätzlicher Weise die Notwendigkeit, sich am Protest zu beteiligen, abgesprochen („*es ergibt keinen Sinn dass du dahin gehst*“). Hannah zieht daraus für sich mögliche weitreichende Konsequenzen und erwägt die Schule zu wechseln. Analog zum obigen Ausschnitt, in dem die Grundschüler*innen mit einer „Agency“ ausgestattet werden, die es ihnen ermöglicht, ihren Eltern bei den klimaschädlichen Praktiken ‚auf die Finger zu sehen‘, stattet sich auch Hannah mit der Handlungsmächtigkeit aus, selbstbestimmt die Schule zu verlassen, die offenkundig nicht mit den politischen Anliegen der Schüler*innenprotestbewegung vereinbar ist.

## Schluss

Erste Ergebnisse der explorativ angelegten ethnographischen Studie zur Schüler*innenprotestbewegung „Fridays-for-Future“ weisen auf die im medialen und wissenschaftlichen Diskurs – vermutlich auch aufgrund der quantitativen Unterrepräsentanz von Grundschüler*innen – bislang vernachlässigte Bedeutung der Grundschule als Bildungsinstitution und der Grundschüler*innen als Partizipand*innen hin. Die mit der Studentenbewegung der 1960er-Jahre aufkommende Rede vom „Marsch durch die Institutionen“ erfährt mit der Schüler*innenprotestbewegung eine Wiederbelebung in neuem Gewand, da es nicht um die Zerschlagung der Institutionen, sondern um eine Allianz und Kooperation geht. In Abgrenzung zu gegenteiligen Befunden, wonach der Protest von Schüler*innen darauf gründet, dass gesellschaftliche Institutionen (wie die Schule) die Anliegen der protestierenden Schüler*innenschaft nur defizitär verwirklichen (vgl. u. a. Sutterlüty und Mühlbacher 2018), wird die Grundschule zum ‚verlängerten Arm‘ der Schüler*innenprotestbewegung erklärt, da sie aus Sicht der protestierenden Schüler*innen eine bedeutsame Weichenstellung mit Blick auf die Einübung in klimaprotektive Alltagspraktiken darstellt. Die Wirkmächtigkeit der Grundschule wird dabei von den jugendlichen Aktivistinnen jedoch nicht explizit an eigene schulbiographische Erfahrungen der Grundschulzeit geknüpft, sondern offensichtlich wird mit Projektionen und Erwartungen operiert, die an die Grundschule herangetragen werden. In den Aussagen der Schülerinnen spiegelt sich wider, dass die Grundschule an den Alltagspraktiken anzusetzen habe, die curricular als Unterrichtsthemen (wie zum Beispiel Mülltrennung) thematisch werden – um auch etablierte Alltagspraktiken transformieren zu können (z. B. mit dem Auto zur Schule fahren). Dabei kristallisiert sich heraus, dass es offensichtlich Grundschulen mit einem spezifischen pädagogischen Profil („Klimaschule“, „Draußenschule“) sind, die als ‚Komplizen‘ der Schüler*innenprotestbewegung ins Auge gefasst werden. Inwieweit Grundschüler*innen und Grundschullehrkräfte solcher mit einem spezifischen pädagogischen Profil ausgestatteter Schulen diese Auffassungen teilen bzw. bereits in ihr didaktisches Handeln überführt haben, wäre zukünftig noch genauer zu untersuchen. Vor diesem Hintergrund ließe sich zusammenfassend konstatieren, dass sich der Protest der „Fridays-for-Future-Bewegung“ nicht explizit gegen die Institution (Grund‑)Schule richtet, was ihre Form des *Schulstreiks* von einem *Bildungsstreik* unterscheidet. Gleichzeitig würde es zu kurz greifen, der Protestbewegung eine Schulkonformität zu unterstellen, denn gleichwohl die Schüler*innenprotestbewegung nicht unmittelbar gegen die Bedingungen der Schule aufbegehrt, setzt sie jedoch in subversiver Weise punktuell die gesetzliche Schulpflicht außer Kraft, die nicht nur irgendeine Vorschrift ist. Vielmehr wird mit dem öffentlichen Außerkraftsetzen dieser formalen Erwartung an die Mitgliedschaft als Schüler*in, die schulische Ordnung insgesamt in Frage gestellt, was auch die besondere Wirkung des Protests ausmacht (vgl. Kühl 2005). Unter Rekurs auf Luhmann (1964, S. 63) wird mit der Verletzung der Mitgliedschaftsregel „gegen das System und gegen alle formalen Erwartungen“ rebelliert. Diese Infragestellung der institutionalisierten schulischen Zwangsteilnahme verhilft den Schüler*innen zu einem Autonomiegewinn jenseits der in der Schule „simulierten und verordneten Autonomie“ (Helsper und Lingkost 2004, S. 224). Die Verletzung der gesetzlichen Schulpflicht ermöglicht eine Partizipation jenseits pädagogisch gerahmter Arrangements – wie es etwa auch das Ziel einer Erziehung zur Demokratie bei Dewey (1964) ist. Den Schüler*innen dient die Beteiligung am Protest als *demokratisches Laboratorium*, mit dessen Hilfe ein demokratischer Habitus potenziell eingeübt werden kann, der über die Schule hinausverweist (Edelstein 2007). Anders als bei den institutionalisierten Mitbestimmungsgremien in der (Grund‑)Schule besteht keine offizielle Verpflichtung an den Protestpraktiken zu partizipieren und in der Regel sind es auch nicht Erwachsene, welche die Partizipation organisieren, womit die asymmetrische Erwachsenen-Kind-Differenz aufgehoben und innerschulische bzw. der Grammatik der Schule eingeschriebene Partizipationshindernisse (vgl. oben 2.) potenziell überwunden werden können. Damit ließe sich die These aufstellen, dass sich eine demokratiebildende Grundschule (vgl. Backhaus et al. 2008) – für deren Realisierung Partizipation eine wesentliche Voraussetzung darstellt – gerade nicht nur *im Unterricht* durch die Etablierung innerschulischer Mitbestimmungsgremien zu erkennen gibt, sondern auch und vor allem in anderen *außerschulischen* Zusammenhängen zeigt (wie beim Protest auf der Straße). Außerschulische Erfahrungen demokratischen Handelns können somit die schulischen, limitierten Partizipationsoptionen erweitern und vertiefen, indem die Schüler*innen ihre formale Mitgliedschaft als Schüler*in punktuell aufkündigen und zu Partizipand*innen einer Solidargemeinschaft werden. An dieser Stelle soll jedoch der Romantisierung der Partizipationsmöglichkeiten im Kontext der Schüler*innenprotestbewegung keineswegs Vorschub geleistet werden: Denn wie sich in den Interviews andeutet, bleibt der Partizipationsstatus der Grundschüler*innen im Kontext der Schüler*innenprotestbewegung ambivalent: Sie werden als Partizipand*innen der Bewegung explizit adressiert und nehmen in exponierter Position an den Protestpraktiken teil, werden jedoch offensichtlich – aufgrund ihres Alters – mehr als *zukünftige*, denn als *gegenwärtige* Demokrat*innen betrachtet. Um hier empirisch zuverlässige Aussagen über die Partizipation der Grundschüler*innen an der Schüler*innenprotestbewegung treffen zu können, müssten jedoch stärker noch die „Stimmen“ der protestierenden Grundschüler*innen zu Wort kommen – was im Rahmen des laufenden Projekts auch angestrebt wird. Für die Verschränkung der außerschulischen und schulischen Partizipationspraktiken sind zudem weitere empirische Forschungen anzustreben, die an der Schnittstelle von Grundschul- und Kindheitsforschung angesiedelt sind.

Richtet man den Blick auf die mediale Darstellung des Schüler*innenprotests zeigt sich, dass die Schüler*innenprotestbewegung bisweilen eine *Delegitimierung durch Infantilisierung *erfährt, indem ihnen vorgeworfen wird, Kinder und Jugendliche zu sein, welche den Argumenten der Erwachsenen noch nicht ‚gewachsen‘ seien und deren primäres Anliegen es sei, die Schule schwänzen zu wollen (von Lucke 2019). Damit wird ihnen ein politisch motiviertes Protestverhalten abgesprochen. Gerade hier lohnt es sich für zukünftige Forschungen noch genauer anzusetzen und die medialen und pädagogischen Konstruktionen des *Kindes als politisches Subjekt* in den Blick zu nehmen, ohne dabei die durchaus auch problematischen Verstrickungen zu vernachlässigen, die mit der Politisierung (grundschul-)pädagogischer Praxis verbunden sind^5^. Dabei wäre auch der Partizipationsbegriff kritisch zu befragen, den eine „Aura des Positiven“ (Feichter 2017, S. 38) umgibt, da er – und dies hat er mit anderen pädagogischen Begriffen gemein – mit einer „Aktivismus-Rhetorik“ (Reichenbach 2004) verbunden wird, die ihn politisch in der Disziplin zum Erfolg verhilft, jedoch von seinen Schwächen absieht. Als gewinnbringend könnte es sich erweisen, statt Partizipation (weil es pädagogisch gewünscht erscheint) als normatives Ideal vorauszusetzen, stärker noch die *Praktiken der Partizipation* empirisch in den Blick zu nehmen, die sich im Kontext der Grundschule als Ort und Erfahrungsraum politischer Bildung, aber auch jenseits davon (bspw. bei Schüler*innenprotesten) außerschulisch vollziehen können.

## Footnotes

1 An dieser Stelle sei allen beteiligten Studierenden herzlich gedankt, die im Rahmen des Lehrforschungsprojekts an der Erstellung der Beobachtungsprotokolle, Interviewtranskripte und rekonstruktiven Analysen mitgewirkt haben.

2 Um Missverständnissen vorzubeugen: Die hier präsentierten Ergebnisse fokussieren sich auf die Aussagen bzw. erzählten Praktiken jugendlicher Aktivistinnen und ihren Deutungen zur Grundschule, was jedoch nicht als methodischer Makel (i. S. einer zu dünnen Datenbasis und fehlenden Berücksichtigung der „Stimmen“ der Kinder) zu interpretieren ist, sondern empirische Wirklichkeit abbildet. Aus der quantitativen Unterrepräsentanz und dem „Minoritätenstatus“ der Grundschulkinder lässt sich jedoch nicht vorschnell eine Aussage über die tatsächliche Partizipation an den Protestpraktiken ableiten. Genau hier knüpft das Projekt an und fokussiert auf die subtilen Praktiken, wobei Kinder als Partizipand*innen von Praktiken (Bollig und Kelle 2014) entworfen werden. Eine solche Perspektive ermöglicht es, den relationalen Charakter (Kinder als *Teil *der Schüler*innenprotestbewegung) zu berücksichtigen und zu einer differenzierten Betrachtungsweise von Partizipation zu gelangen, die jenseits eines politisch motivierten Partizipationsideals, Variabilität, Abstufungen und unterschiedliche Formen von Partizipation zulässt.

3 Mit dem Begriff des „generationalen Umordnens“ soll hier zum Ausdruck gebracht werden, dass sich eine Art ‚Rollenumkehrung‘ zwischen Erwachsenen und Kindern vollzieht, wobei letztere in der Verantwortung stehen, die Eltern auf ein mögliches ökologisches Fehlverhalten hinzuweisen.

4 Die „Draußenschule“ ist ein schulpädagogisches Konzept, welches sich vor allem seit den 1990er-Jahren in Norwegen (Uteskole) und Dänemark (Udeskole) entwickelt hat. Das Konzept beinhaltet dabei, dass die Schulklassen einmal pro Woche den Klassenraum verlassen und Naturräume aufsuchen.

5 Reichenbach (2016) zeigt etwa am Beispiel des „Frontalunterrichts“ auf, wie der im schulpädagogischen Diskurs Verwendung findende Begriff politisch aufgeladen wird, indem ihm etwas *Konservatives* und *Martialisches *angeheftet wird.
